# Inhibition of Midkine Augments Osteoporotic Fracture Healing

**DOI:** 10.1371/journal.pone.0159278

**Published:** 2016-07-13

**Authors:** Melanie Haffner-Luntzer, Julia Kemmler, Verena Heidler, Katja Prystaz, Thorsten Schinke, Michael Amling, Anna Kovtun, Anna E. Rapp, Anita Ignatius, Astrid Liedert

**Affiliations:** 1 Institute of Orthopedic Research and Biomechanics, University Medical Center Ulm, Ulm, Germany; 2 Institute of Osteology and Biomechanics, University Medical Center Hamburg-Eppendorf, Hamburg, Germany; Nanjing Medical University, CHINA

## Abstract

The heparin-binding growth and differentiation factor midkine (Mdk) is proposed to negatively regulate osteoblast activity and bone formation in the adult skeleton. As *Mdk*-deficient mice were protected from ovariectomy (OVX)-induced bone loss, this factor may also play a role in the pathogenesis of postmenopausal osteoporosis. We have previously demonstrated that Mdk negatively influences bone regeneration during fracture healing. Here, we investigated whether the inhibition of Mdk using an Mdk-antibody (Mdk-Ab) improves compromised bone healing in osteoporotic OVX-mice. Using a standardized femur osteotomy model, we demonstrated that Mdk serum levels were significantly enhanced after fracture in both non-OVX and OVX-mice, however, the increase was considerably greater in osteoporotic mice. Systemic treatment with the Mdk-Ab significantly improved bone healing in osteoporotic mice by increasing bone formation in the fracture callus. On the molecular level, we demonstrated that the OVX-induced reduction of the osteoanabolic beta-catenin signaling in the bony callus was abolished by Mdk-Ab treatment. Furthermore, the injection of the Mdk-Ab increased trabecular bone mass in the skeleton of the osteoporotic mice. These results implicate that antagonizing Mdk may be useful for the therapy of osteoporosis and osteoporotic fracture-healing complications.

## Introduction

Osteoporosis affects 200 million women worldwide; one major risk factor is the decline in estrogen after menopause [1, 2]. The disease is characterized by a loss of bone mass and a destruction of bone microarchitecture, resulting in increased fracture risk [3]. Osteoporotic fractures will be experienced by 33% of women aged over 50 years [4]. Clinical studies reported a decreased regeneration potential and a prolonged healing time in osteoporotic patients [5]. This was confirmed by numerous experimental studies in small and large animals [6–9]. However, the molecular pathomechanisms of osteoporotic bone healing are poorly understood. One growth factor that may be a promising target molecule during delayed bone healing is midkine (Mdk), because it is proposed to be involved in the development of postmenopausal osteoporosis [10]. Mdk belongs to the family of heparin-binding growth and differentiation factors [11] and is expressed during embryonic tooth and limb development [12, 13]. Mdk is proposed to be a negative regulator of bone remodeling, because adult *Mdk*-deficient mice display an increased bone formation rate [10]. Bone formation in response to mechanical load was also significantly enhanced in the absence of Mdk [14]. The negative effects of Mdk on bone formation appeared to be mediated by the inhibition of Wnt/beta-catenin signaling in osteoblasts [14], a crucial pathway for osteoblast function [15]. We recently demonstrated that treatment with an antagonizing Mdk-antibody (Mdk-Ab) significantly accelerated fracture healing in mice, presumably by increasing beta-catenin signaling in osteoblasts [16]. We also found a fracture-induced increase in Mdk serum levels, which was attenuated by the Mdk-Ab. Because it was shown previously that *Mdk* is an estrogen-responsive gene and that Mdk expression is enhanced in the murine postmenopausal diabetic kidney [17], we hypothesized that Mdk may also be involved in the pathogenesis of compromised bone healing after estrogen withdrawal. The present study demonstrated that Mdk serum levels were significantly increased after fracture, particularly in osteoporotic mice, and that treatment with an Mdk-Ab abolished the negative influence of ovariectomy (OVX) on bone healing. Moreover, trabecular bone mass was enhanced in OVX-mice which received Mdk-Ab. These results imply that antagonizing Mdk may be a potential therapeutic strategy to enhance fracture healing in patients with osteoporosis.

## Materials & Methods

### Study approval

All animal experiments were in compliance with international regulations for the care and use of laboratory animals (ARRIVE guidelines and EU Directive 2010/63/EU for animal experiments) with the approval of the Local Ethical Committee (No. 1079, Regierungspräsidium Tübingen, Germany).

### Animal experiments

Female C57BL/6J mice were provided by the University of Ulm and were maintained in groups of two to four animals per cage (370 cm^2^) on a 14 h light and 10 h dark circadian rhythm with water and food *ad libitum*. Three-month-old mice underwent a bilateral sham-operation or OVX as described previously [18]. Osteotomy was performed 8 weeks after sham/OVX as published previously [19, 20]. Briefly, all mice received a standardized osteotomy at the midshaft of the right femur using a 0.4 mm Gigli saw (RISystem, Davos, Switzerland) stabilized using an external fixator (axial stiffness of 3.0 N/mm, RISystem). All mice were fed a phytoestrogen-free diet for the entire experimental period. The mice were randomly assigned to the treatment groups. The Mdk-Ab (mouse anti-human Mdk antibody, cross-reactive to murine Mdk; provided by Cellmid Ltd, Sydney, Australia) was administered subcutaneously at 25 mg/kg twice weekly for 3 weeks to half of the animals, application was initiated directly after surgery. The control animals were treated in parallel using the vehicle phosphate-buffered saline (PBS), because our previous study reported no differences in the fracture healing process between PBS- or control immunoglobulin G (IgG)-treated mice [16]. Mice were sacrificed 3, 10 or 23 days after surgery using carbon dioxide (n = 6–7 per group at each time point). These time points were chosen to assess the inflammatory phase, the endochondral ossification phase and the bony remodeling stage of fracture healing. Blood samples were collected from mice pre-operatively and on days 3, 10 and 23. The fractured and intact femurs as well as the lumbar vertebral bodies were removed from all mice for further analysis.

### Serum analysis

The serum Mdk protein level was determined using a human Mdk enzyme-linked immunosorbent assay kit (provided by Cellmid Ltd) as described in the manufacturer’s protocol, which was cross-reactive with murine Mdk.

### Biomechanical testing

Biomechanical testing of the intact and fractured femurs of the mice sacrificed at day 23 was performed using a nondestructive 3-point-bending test as described previously [19]. Briefly, after removal of the fixator, the bone was loaded in a materials testing machine and the load and deflection were recorded (Zwick Roell, Ulm, Germany). The flexural rigidity of the fracture callus was calculated using the slope of the load-deflection curve. The relative flexural rigidity of the fractured femur was calculated as the ratio between the fractured and intact femur of the same mouse.

### Micro-computed tomography (μCT)

Femurs and vertebral bodies were analyzed using a μCT scanning device (Skyscan 1172, Kontich, Belgium) operating at a voxel resolution of 8 μm (50 kV, 200 mA). Four volumes of interest (VOIs) were determined for μCT analysis: VOI 1 (fracture callus) covered the periosteal callus between the two inner pinholes. VOI 2 (intact cortical bone) covered the area from 80 μm proximal to 80 μm distal from the middle of the diaphysis of the intact femur. The starting point for VOI 3 (intact trabecular bone) was 200 μm proximal to the metaphyseal growth plate of the intact femur and the endpoint was 280 μm proximal from the starting point. VOI 4 covered the trabecular region of the second lumbar vertebral body. Tissue and bone mineral density (TMD and BMD) were assessed using two phantoms with a defined hydroxyapatite density (250 mg/cm^3^ and 750 mg/cm^3^) within each scan. The BMD of the fracture callus was evaluated without a threshold, whereas the TMD of the cortical bone was determined using a global threshold of 642 mg hydroxyapatite/cm^3^. The TMD of the trabecular bone was evaluated using a global threshold of 395 mg hydroxyapatite/cm^3^ as described previously and in accordance with the American Society for Bone and Mineral Research (ASBMR) guidelines for μCT analysis [18, 21].

### Histomorphometry of undecalcified femora

The amounts of bone, cartilage and fibrous tissue in the whole callus between the two inner pin holes were determined using undecalcified histological sections of fractured femurs explanted at day 23 as described previously [20]. Briefly, femurs were fixed in 4% formalin, dehydrated in an ascending ethanol series and embedded in methyl methacrylate. Sections of 7 μm were prepared and stained using Giemsa for histomorphometric analysis. The amounts of bone, cartilage and fibrous tissue were determined using image-analysis software (Leica MMAF 1.4.0 Imaging System, Leica). Osteoblasts and osteoblast surface were determined using sections stained with Toluidine Blue and analyzed under 400-fold magnification. Tartrate-resistant acid phosphatase staining was used to identify osteoclast numbers and surface. All parameters were determined using the Osteomeasure system (Osteometrics, Decatur, USA). Region of interest (ROI) in the fracture callus was a 1.5 mm x 0.35 mm rectangular region in the middle of the periosteal callus. ROI in the intact femur was the trabecular region of the proximal metaphysis, starting 200 μm proximal to the metaphyseal growth plate.

### Histomorphometry of decalcified femora and immunohistochemistry

Femurs of mice sacrificed 10 days post-surgery were fixed in 4% formalin, decalcified using 20% ethylenediaminetetraacetic acid (pH 7.2–7.4) for 10–12 days and embedded in paraffin after dehydration in an ascending ethanol series. Longitudinal sections of 7 μm thickness were prepared and stained using Safranin O for tissue quantification. Immunohistochemical staining of total and active beta-catenin was performed using the following antibodies: polyclonal rabbit anti-mouse beta-catenin antibody (AB19022, EMD Millipore Corporation, Merck, Darmstadt, Germany), monoclonal rabbit anti-mouse active beta-catenin antibody (#8814, Cell Signaling, Frankfurt am Main, Germany), horse-radish peroxidase-conjugated streptavidin (Zytomed Systems, Berlin, Germany), and goat anti-rabbit IgG (Invitrogen, ThermoFisher Scientific, Waltham, USA). Species-specific non-targeting immunoglobulins were used as isotype controls. 3-Amino-9-ethylcarbazol (Zytomed Systems) was used as the chromogen and the sections were counterstained using hematoxylin (Waldeck, Münster, Germany) as described previously [20]. Quantification of the positively stained regions for beta-catenin in the whole fracture callus was performed using the image analysis software Adobe Photoshop CS4 (Adobe, Dublin, Ireland) as described previously [16].

### Statistics

Sample size was calculated based on a previous fracture-healing study for the main outcome parameter flexural rigidity in the fractured femur (power: 80%, alpha = 0.05) [18]. The results of the present study were analyzed for significance using the Mann-Whitney-U or Kruskal-Wallis test with Dunn’s post hoc test. All results are presented as box plots with median, first and third quartiles and maximum and minimum values. Values of p<0.05 were considered to be statistically significant.

## Results

### Mdk-Ab treatment abolished the negative effect of OVX on fracture healing

**Fig 1:**
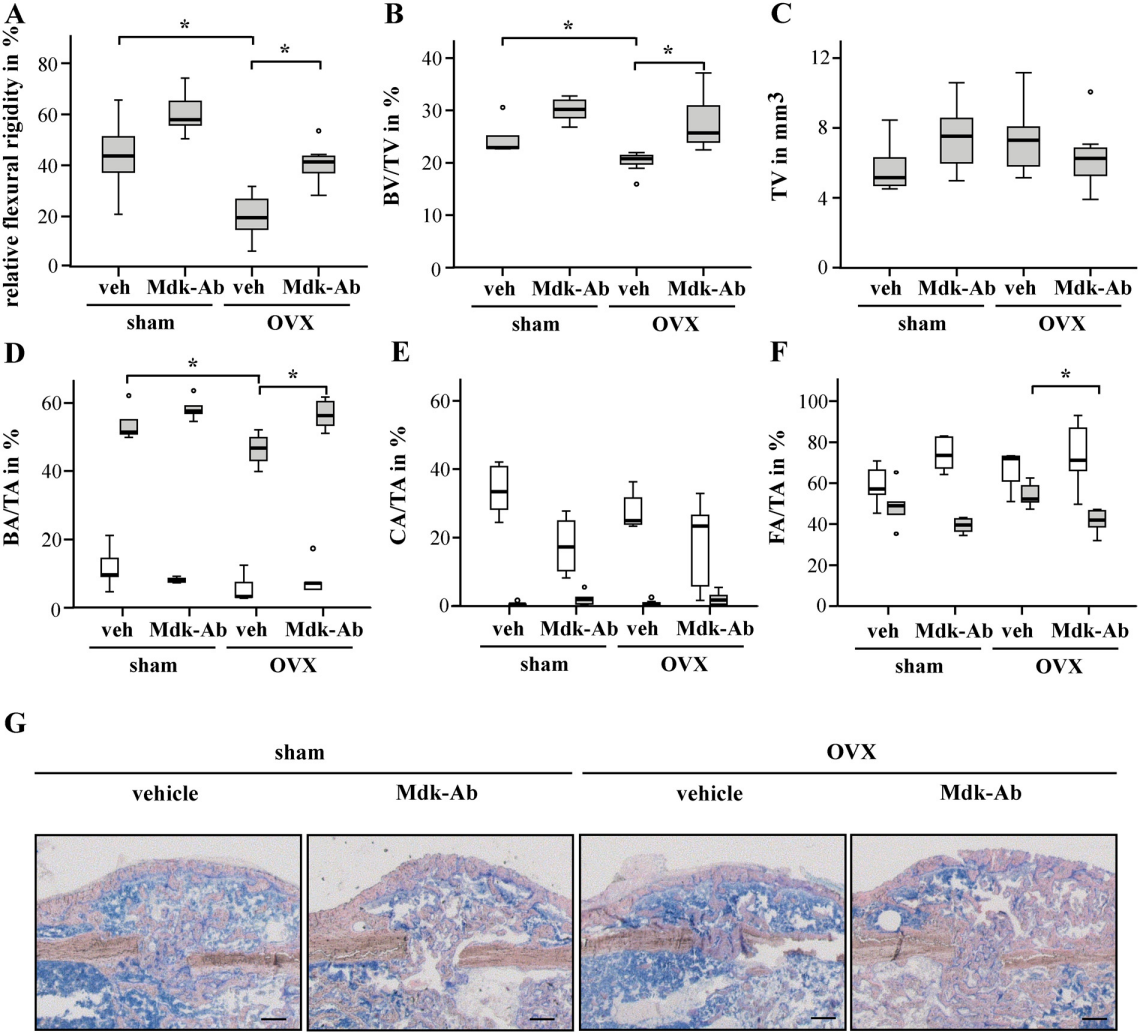
Midkine-antibody (Mdk-Ab) treatment accelerated osteoporotic fracture healing. Biomechanical, micro-computed tomography (μCT) and histomorphometric analysis of the fractured femurs at day 23. Biomechanical testing: A) relative flexural rigidity of the fractured femur in comparison with intact femur determined by biomechanical testing. Parameters determined by μCT analysis (volume of interest 2): B) bone volume to tissue volume ratio and C) tissue volume (n = 6–7 per group). Parameters determined by histomorphometric analysis of the whole fracture callus at day 10 (white bars) and day 23 (grey bars): D) bone area to tissue area ratio, E) cartilage area to tissue area ratio and F) fibrous tissue area to tissue area ratio. *Significantly different from sham+vehicle or OVX+vehicle group (p<0.05) by Kruskal-Wallis test. (n = 5–7 per group.) G) Representative images of sections from undecalcified femurs at day 23, stained using Giemsa; scale bar: 250 μm.

As expected, OVX delayed fracture healing as demonstrated by a significantly decreased relative flexural rigidity of the fractured femurs of the vehicle-treated mice (Fig 1A). Callus size was unaffected by OVX (Fig 1C), whereas bone volume to tissue volume ratio (BV/TV) was significantly decreased in the calli of OVX mice (Fig 1B). This was confirmed by histomorphometric analysis, showing a decreased percentage of bone area on day 23 (Fig 1D). The decreased bone formation due to OVX might be based on the significantly decreased osteoblast surface and significantly increased osteoclast numbers in the fracture callus suggesting decreased osteoblast activity and increased osteoclast resorption (Table 1). There were no significant differences between non-OVX and OVX mice on day 10 (Fig 1D–1F).

**Table 1:** Cellular parameters in the fracture callus and the intact femur at day 23.

	fracture callus				intact femur			
	NOb/BPm (1/mm)	ObS/BS (%)	NOc/BPm (1/mm)	OcS/BS (%)	NOb/BPm (1/mm)	ObS/BS (%)	NOc/BPm (1/mm)	OcS/BS (%)
**sham vehicle**	16.5 ± 4.0	21.7 ± 5.2	3.2 ± 0.8	5.0 ± 1.0	7.5 ± 3.0	8.8 ± 1.6	1.2 ± 0.1	1.3 ± 0.1
**sham Mdk-Ab**	17.6 ± 4.4	26.9 ± 5.7	2.5 ± 0.2	3.9 ± 0.8	9.1 ± 2.2	10.7 ± 1.6	1.2 ± 0.4	1.3 ± 0.3
**OVX vehicle**	13.3 ± 2.8	16.4 ± 3.6^a^	4.5 ± 1.9^a^	5.6 ± 2.5	4.5 ± 0.8^a^	6.5 ± 1.3^a^	2.7 ± 1.6^a^	4.0 ± 2.1^a^
**OVX Mdk-Ab**	19.4 ± 3.4^b^	28.0 ± 5.6^b^	1.9 ± 0.5^b^	2.7 ± 0.6^b^	6.4 ± 0.3^b^	8.4 ± 0.4^b^	2.0 ± 0.3	3.2 ± 0.6

NOb/BPm: number of osteoblasts per bone perimeter; ObS/BS: osteoblast surface relative to the bone surface; NOc/BPm: number of osteoclasts per bone perimeter; OcS/BS: osteoclast surface relative to the bone surface; n = 5–7 per group presented as the mean ± standard deviation.

^a^ = p<0.05 for effect of ovariectomy (comparison between sham vehicle and OVX vehicle groups);

^b^ = p<0.05 for effect of midkine-antibody (comparison between sham vehicle and sham Mdk-Ab, or OVX vehicle and OVX Mdk-Ab, respectively).

Mdk-Ab treatment tended to increase the relative flexural rigidity in the sham-operated group and significantly in the OVX group (Fig 1A). μCT analysis of the fracture calli demonstrated an enhanced BV/TV after antibody treatment (Fig 1B). Callus size was unaffected by Mdk-Ab treatment (Fig 1C). Histomorphometric analysis showed no significant differences between the treatment groups at day 10 after surgery, only a tendency to a decreased cartilage area in Mdk-Ab-treated mice (Fig 1D–1F). In contrast, by day 23, the decreased bone content in the fracture callus of OVX mice was abolished by Mdk-Ab treatment (Fig 1D and 1G). Similarly, fibrous tissue content was significantly decreased in the OVX mice after Mdk-Ab treatment (Fig 1F and 1G). Additionally, Mdk-Ab treatment significantly increased the number and surface of osteoblasts and decreased the number and surface of osteoclasts in the fracture callus of OVX mice (Table 1).

### Mdk serum levels were increased after fracture

We determined Mdk serum levels 0, 3, 10 and 23 days after fracture (Table 2). None of the mice had a detectable systemic Mdk concentration at day 0. Fracture increased the Mdk serum levels in both sham-operated and OVX mice after 3 days. However, the Mdk serum level remained elevated only in OVX mice until day 23. At day 10, Mdk-Ab treatment significantly decreased Mdk serum levels in OVX animals.

**Table 2:** Midkine (Mdk) serum levels during fracture healing in 3-month-old sham-operated and ovariectomized (OVX) mice in pg/ml.

	treatment			
	sham		OVX	
days after operation	vehicle	Mdk-Ab	vehicle	Mdk-Ab
d0	n.d.		n.d.	
d3	38.6 ± 44.6	15.1 ± 33.9	67.9 ± 45.7	37.8 ± 43.6
d10	n.d.	n.d.	61.7 ± 43.6^a^	n.d.^b^
d23	n.d.	n.d.	31.0 ± 28.2	n.d.

n.d.: not detectable. d0: pre-operation value. (n = 5–7 per group presented as the mean ± standard deviation.)

^a^ = p<0.05 for effect of ovariectomy (OVX);

^b^ = p<0.05 for effect of Mdk-antibody (Mdk-Ab).

### Mdk-Ab treatment abolished OVX-induced decreased beta-catenin expression

**Fig 2:**
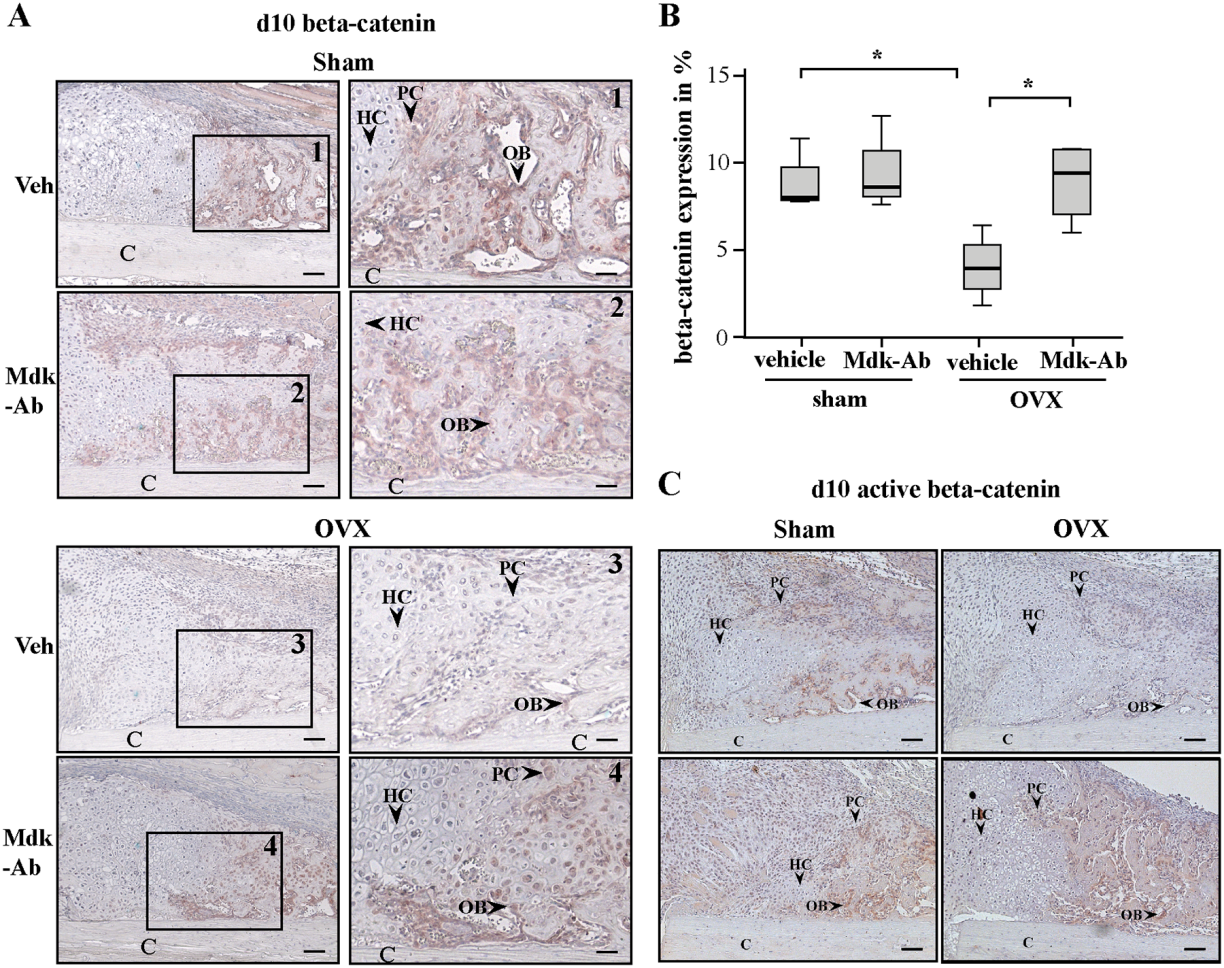
Beta-catenin-positive area was decreased after ovariectomy (OVX) and increased after midkine-antibody (Mdk-Ab) treatment. A) Sections of fractured femurs from four mice of each group were stained for beta-catenin and counterstained using hematoxylin. Representative images of the periosteal callus proximal to the osteotomy gap at day 10 after fracture are shown. Scale bar left column: 100 μm. Scale bar right column: 50 μm. B) Quantification of the beta-catenin-positive area in %. (n = 4 per group.) *Significantly different from sham+vehicle or OVX+vehicle group (p>0.05). C) Sections of fractured femurs from four mice of each group were stained for active beta-catenin and counterstained using hematoxylin. Representative images of the periosteal callus proximal to the osteotomy gap at day 10 after fracture are shown. Scale bar: 100 μm. C = cortex; HC = hypertrophic chondrocytes; OB = osteoblasts; PC = proliferating chondrocytes. Upper row: vehicle treatment; lower row: Mdk-Ab treatment.

We were next interested in the molecular mechanism behind the increased bone formation in the fracture callus of Mdk-Ab-treated mice. Therefore, we stained callus sections for beta-catenin expression. Beta-catenin expression was mainly found in proliferative chondrocytes at the cartilaginous callus and in osteoblasts at the bony fracture callus, whereas hypertrophic chondrocytes were negative (Fig 2A). OVX significantly decreased the beta-catenin-positive area in the bony fracture callus at day 10 (Fig 2A and 2B). Mdk-Ab treatment did not alter the beta-catenin expression in sham-operated mice, whereas there was a significant increase in the beta-catenin-positive area in Mdk-Ab-treated OVX mice. Additionally, we stained the sections for the active form of beta-catenin (Fig 2C). The osteoblasts at the bony fracture callus were found to highly express the active beta-catenin form. Mdk-Ab increased the expression in treated OVX mice.

### Mdk-Ab treatment increased bone formation in the intact femur and the vertebral bodies of osteoporotic mice

**Fig 3:**
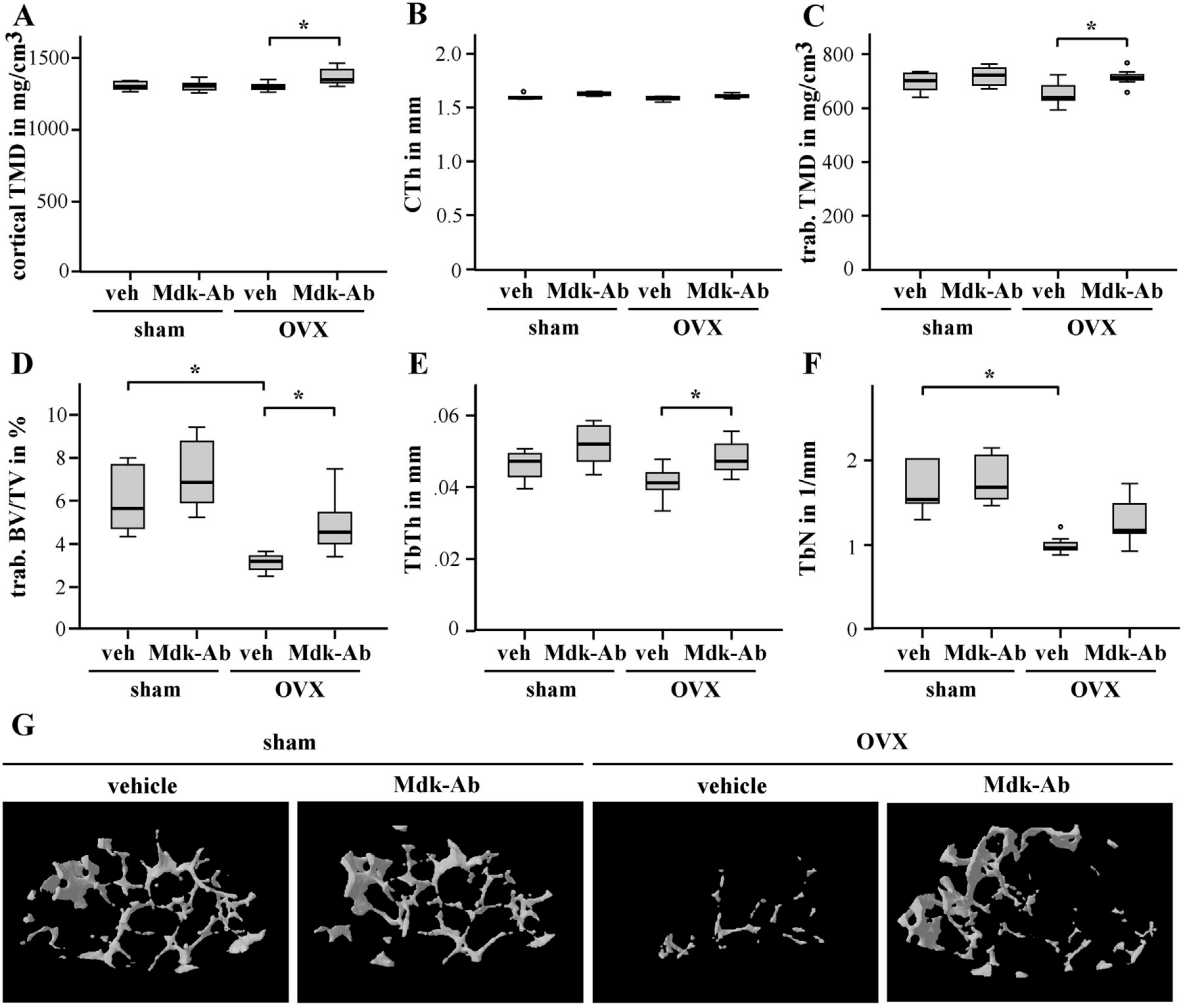
Midkine-antibody (Mdk-Ab) treatment increased the bone content in the intact femur of ovariectomized (OVX) mice after short-time treatment. Micro-computed tomography (μCT) analysis of the cortical bone at the midshaft of the intact femur volume of interest 2 (VOI 2): A) cortical tissue mineral density (TMD) and B) cortical thickness. μCT analysis of the distal part of the intact femur (VOI 3): C) trabecular TMD, D) bone volume to tissue volume ratio, E) trabecular thickness and F) trabecular number. *Significantly different from sham+vehicle or OVX+vehicle group (p<0.05) by Kruskal-Wallis test. (n = 6–7 per group.) G) Three-dimensional reconstructions of the trabecular region of the distal intact femur (VOI 3), representative images are shown.

Antibody treatment increased the cortical TMD in the intact femur of OVX animals (Fig 3A), whereas cortical thickness was unaffected in all groups (Fig 3B). Evaluation of the trabecular region of the intact femur confirmed an osteoporotic phenotype in OVX mice; trabecular BV/TV and number were significantly decreased in vehicle-treated mice, as well as osteoblast number and surface (Table 1). Osteoclast number and surface were increased after OVX.

Mdk-Ab treatment significantly increased trabecular TMD, BV/TV, trabecular thickness, osteoblast numbers and surface in OVX mice (Fig 3C–3G, Table 1). Bone parameters in sham-operated mice were only slightly increased after antibody treatment. There was no influence of the antibody treatment on the osteoclasts in the intact femur.

**Fig 4:**
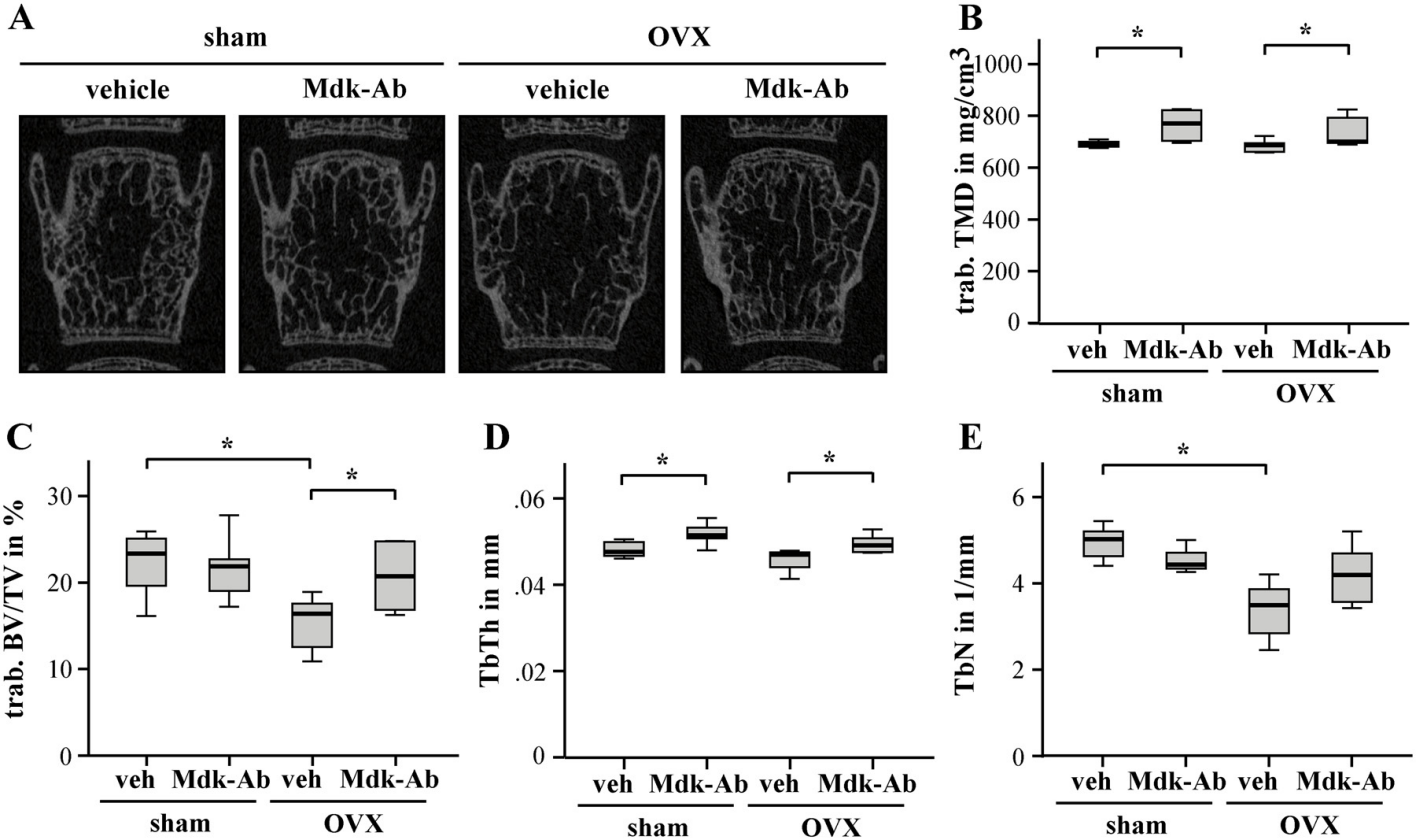
Midkine-antibody (Mdk-Ab) treatment increased the bone content in vertebral bodies of ovariectomized (OVX) mice after short-time treatment. Micro-computed tomography (μCT) analysis of the vertebral bodies (volume of interest 4): A) two-dimensional images of the second caudal vertebral body, representative images are shown. B) Trabecular tissue mineral density, C) trabecular bone volume to tissue volume ratio, D) trabecular thickness and E) trabecular number. *Significantly different from sham+vehicle or OVX+vehicle group (p<0.05) by Kruskal-Wallis test. (n = 6−7 per group).

OVX also led to a decreased trabecular BV/TV and number in the vertebral bodies of vehicle-treated mice (Fig 4A). Mdk-Ab treatment significantly increased the vertebral trabecular TMD, BV/TV and thickness in the OVX animals (Fig 4B–4D). We found a greater impact of the antibody treatment on the trabecular bone of the spine than of the femur, particularly in sham-operated mice; trabecular TMD and thickness were significantly improved by Mdk-Ab treatment, whereas trabecular BV/TV and number remained unchanged in the sham-operated animals (Fig 4C and 4E).

## Discussion

Because osteoporotic patients display an increased risk for fracture healing complications [5], there is a considerable clinical need for treatment strategies to enhance osteoporotic bone regeneration. One promising target molecule may be the growth- and differentiation factor Mdk, because it negatively influences osteoblast activity [14]. We have recently shown that treatment with Mdk-Ab accelerated bone regeneration in adult mice by decreasing the fracture-induced increased level of systemic Mdk [16]. In the present study, we investigated the application of the Mdk-Ab during osteoporotic fracture healing using a mouse model of OVX-induced osteoporosis.

As expected, OVX mice displayed a significantly lower relative flexural rigidity and bone content in the fracture callus compared to non-OVX mice [18, 22, 23]. This was due to decreased osteoblast activity and increased osteoclast numbers in the bony callus. These negative effects of OVX on bone healing were decreased by Mdk-Ab treatment. Therefore, our data indicate that Mdk may be involved in the pathogenesis of delayed healing after estrogen withdrawal. Indeed, we found a fracture-induced increase of Mdk serum levels in both sham and OVX mice, however, OVX mice displayed significantly higher levels and showed a prolonged presence of systemic Mdk until day 23 after fracture. Moreover, it has been previously shown that Mdk is an estrogen-responsive gene and that Mdk expression is enhanced in the murine postmenopausal diabetic kidney [17]. Furthermore, Kondoh et al. demonstrated an increased Mdk expression in osteocytes of osteoporotic mice due to the lack of estrogen receptor α in osteocytes [24]. On the molecular level, beta-catenin signaling appears to be involved in both OVX-induced delayed fracture healing and in the positive effects of the Mdk-Ab treatment. We found that OVX significantly decreased the beta-catenin positively stained area in the fracture callus, as shown previously [18]. There was no significant difference in beta-catenin expression between the treatment groups in sham-operated mice, whereas Mdk-Ab significantly increased the beta-catenin positively stained area of OVX mice. In agreement with that finding, it has been shown previously that Mdk acts as a negative regulator of beta-catenin signaling in osteoblasts [14] by decreasing the phosphorylation of the Wnt/beta-catenin receptor low-density lipoprotein receptor-related protein 6 (LRP6) and that antagonizing Mdk increased the expression of beta-catenin in the bony fracture callus of middle-aged mice [16]. Therefore, enhanced beta-catenin signaling after Mdk-Ab administration appears to be the cause for the accelerated bone formation and, therefore, improved fracture healing in OVX mice, because it was shown previously that Wnt/beta-catenin signaling activation significantly accelerated fracture repair in mice, rats and nonhuman primates [25–27]. Comparing the results of the present study to our previous study using 9-month-old mice [16], we noticed that the effects of the Mdk-Ab treatment were more prominent in the middle-aged mice than in the younger sham-operated mice used in the present study (3-month-old). The adult mice showed higher serum Mdk levels compared to the young mice [16], explaining the minor effects of the treatment on the control mice in the present study. Indeed, it was shown previously that the positive effects of *Mdk*-deficiency were more pronounced in 12- and 18-month-old mice than in 4-month-old mice [10]. This indicates that age can also influence the level of systemic Mdk, thereby affecting fracture healing. Indeed, it was shown previously, that aged mice displayed delayed bone healing [28–31]. Wnt/beta-catenin signaling appears to play a role during ageing-induced disturbed bone regeneration, because beta-catenin expression was demonstrated to be more intense in the fracture callus of young mice compared to aged mice [32]. Therefore, it might be a limitation of the present study that young mice were used, because postmenopausal osteoporosis mainly occurs in aged patients.

Because *Mdk*-deficient mice exhibited increased trabecular bone formation in the vertebral bodies and were protected from OVX-induced trabecular bone loss [10], we also investigated the effects of the Mdk-Ab on intact bone. Mdk-Ab treatment increased both the cortical and trabecular bone mineralization, and the bone mass in the intact femur of OVX mice, whereas no significant effects were observed in the sham-operated mice. In contrast, we observed positive effects in both Mdk-Ab-treatment groups on the bone mass and mineralization related to the trabecular bone of the vertebral bodies. This may be due to the greater remodeling activity in the spinal trabecular bone [33]. On the molecular level, it was shown previously that treatment of osteoblasts with recombinant Mdk induced the expression of several genes, which were known to be associated with matrix mineralization and osteoblast differentiation, namely *Dmp1*, *Ank* and *Enpp1* [10]. In particular, *Ank* and *Enpp1* are essential in preventing ectopic tissue mineralization by raising the extracellular level of pyrophosphatase [34]. Therefore, it is reasonable that antagonizing Mdk using Mdk-Ab may induce greater tissue mineralization and osteoblast differentiation. Indeed, we demonstrated significantly increased number and activity of osteoblasts in the intact femur of OVX mice after Mdk-Ab treatment. Our finding that Mdk-Ab increased the amount of trabecular bone even after this short-term treatment indicates that Mdk-Ab may be a possible new candidate for osteoporosis therapy.

In conclusion, the findings of the present study indicate that there is a strong therapeutic potential for the Mdk-Ab to enhance fracture healing in patients with delayed bone healing resulting from postmenopausal osteoporosis.
